# Inpatient remdesivir versus nirmatrelvir-ritonavir in the progression of COVID-19

**DOI:** 10.1017/ash.2023.297

**Published:** 2023-09-29

**Authors:** Dimple Patel, Christopher Mccoy, Kendall Donohoe, Matthew Lee, Howard Gold, Ryan Chapin

## Abstract

**Background:** Nirmatrelvir-ritonavir received emergency use authorization (EUA) for the prevention of progression of COVID-19 in December 2021. Most data supporting this authorization are limited to the outpatient setting in unvaccinated patients, and high-quality head-to-head comparisons to other antivirals such as remdesivir are lacking. Patients at high risk of disease progression, such as advanced age, smokers, and those with cardiovascular disease, diabetes, obesity, or cancer continue to be admitted to acute-care settings for various indications, and some are incidentally found to have mild COVID-19. The objective of this project was to compare rates of progression of mild-to-moderate COVID-19 for inpatients treated with remdesivir versus nirmatrelvir-ritonavir. **Methods:** This study was a single-center, retrospective cohort study that included patients aged ≥18 years with PCR-confirmed SARS-CoV-2 infection who were initiated on nirmatrelvir-ritonavir within 5 days or remdesivir within 7 days of symptom onset between June 2022 and August 2022. The primary outcome was the worsening of symptoms via the WHO ordinal clinical severity scale for COVID-19. Secondary outcomes included escalation of care or readmission due to COVID-19, discharge prior to treatment completion, and any adverse drug reactions (ADRs). Within our institutional guidelines, prior approval is needed for COVID-19 treatment through collaboration between the primary team and antimicrobial stewards. Nirmatrelvir-ritonavir is the preferred agent for both in- and outpatients unless the patient had drug interactions or lack of enteral access, in which case remdesivir was considered. **Results:** In total, 58 patients were screened and 50 patients were included, 25 patients in each arm. Most were non-Hispanic, white males with at least 1 comorbidity. Compared to the remdesivir arm, the nirmatrelvir-ritonavir arm had more patients with at least a primary COVID-19 vaccine (44% vs 34%). Also, 88% of patients in each arm had a baseline ordinal score of 4, and 12% had a score of 5. Ordinal score changes between the start and end of therapy were similar between groups, and neither had an increase in oxygen requirements (Fig. 1). No readmissions were due to COVID-19, and both medications were well tolerated. Refer to Fig. 2 for secondary outcomes. **Conclusions:** Nirmatrelvir-ritonavir and remdesivir showed similar safety and efficacy in the treatment of hospitalized patients with mild-to-moderate COVID-19. Current evidence-based guidelines and treatment costs favor nirmatrelvir-ritonavir for patients who can receive this drug.

**Figure:**
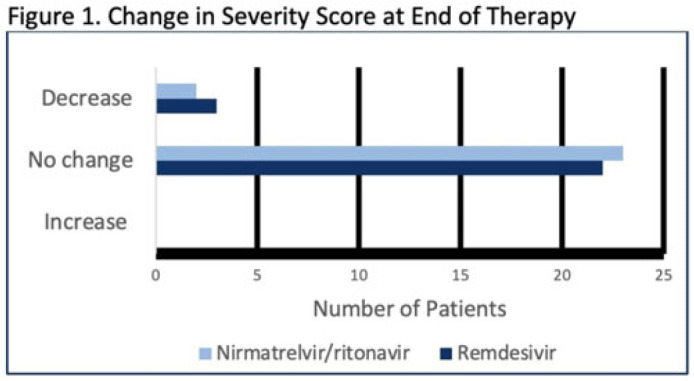

**Figure:**
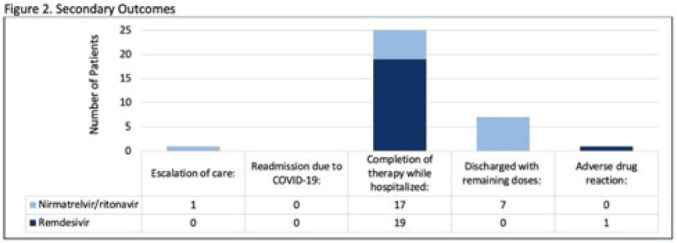

**Disclosures:** None

